# Fault Isolation and Estimation in Networks of Linear Process Systems

**DOI:** 10.3390/e25060862

**Published:** 2023-05-28

**Authors:** Wijaya Kurniawan, Katalin M. Hangos, Lőrinc Márton

**Affiliations:** 1 Department of Electrical Engineering and Information Systems, University of Pannonia, 8200 Veszprem, Hungary; hangos@sztaki.hu; 2 Systems and Control Laboratory, Institute for Computer Science and Control, 1111 Budapest, Hungary; 3 Department of Electrical Engineering, Sapientia Hungarian University of Transylvania, 547367 Corunca, Romania; martonl@ms.sapientia.ro

**Keywords:** process network, fault isolation, disturbance observer, network diagnosis

## Abstract

Fault detection and isolation is a ubiquitous task in current complex systems even in the linear networked case when the complexity is mainly caused by the complex network structure. A simple yet practically important special case of networked linear process systems is considered in this paper with only a single conserved extensive quantity but with a network structure containing loops. These loops make fault detection and isolation challenging to perform because the effect of fault is propagated back to where it first occurred. As a dynamic model of network elements, a two input single output (2ISO) LTI state-space model is proposed for fault detection and isolation where the fault enters as an additive linear term into the equations. No simultaneously occurring faults are considered. A steady state analysis and superposition principle are used to analyse the effect of faults in a subsystem that propagates to the sensors’ measurements at different positions. This analysis is the basis of our fault detection and isolation procedure that provides the position of the faulty element in a given loop of the network. A disturbance observer is also proposed to estimate the magnitude of the fault inspired by a proportional-integral (PI) observer. The proposed fault isolation and fault estimation methods have been verified and validated by using two simulation case studies in the MATLAB/Simulink environment.

## 1. Introduction

Nowadays, we live in a world that is surrounded by networks, e.g., computer networks, transportation networks, social networks, electrical networks, etc. Although they could comprise simple elements, their general large number and interconnections make them an important subclass of complex systems. As a dynamic system, a network presents many theoretical challenges during control or diagnosis method design. There have been many excellent surveys about this in systems and control literature (see, e.g., [1,2,3,4,5,6,7]).

Fault detection and isolation (FDI) is a subfield of control engineering which mainly deals with process monitoring to ensure the safety of production processes. The basic FDI notions are used similarly as presented in [8]. The *fault* is considered an unexpected variation of some process or environmental variable that could yield unacceptable changes in the process behaviour. During system model construction, *additive faults* are assumed and the faults are defined as fictive inputs. By *fault detection*, we mean a decision on the presence of any fault or the absence of all faults. Meanwhile, *fault isolation* is related to the localization of a fault. During *weak fault isolation*, the faults are assumed to happen one at a time. By *fault estimation*, we mean the method that can determine the steady state value of the additive faults based on the available measurements.

Although it is crucial to ensure safe operation in networks of process systems, the number of works related to FDI in networked systems is still limited [9].

There exist many different techniques that can be applied to solve an FDI problem. The important main categories comprise model based and data-driven methods [10,11,12]. Commonly, model-based methods use dynamic models derived from first principles that rely heavily on the knowledge of basic physical and/or chemical processes. Meanwhile, the current popular data-driven methods rely on data from system operations that are used to train machine learning (ML) or artificial intelligence (AI) data structures to detect and identify faults. However, even though data-driven methods could be easier to implement compared to model-based approaches, their reliability strictly depends on the availability and quality of the data [13].

The FDI problem in industrial networks, such as process networks, presents special challenges. For the sake of energy and/or material efficiency, such networks usually contain loops. Therefore, fault diagnosis is harder to perform as the effect of the fault is propagated back to the subsystem where it first occurred [14,15,16,17,18]. Recent research has approached the fault isolation problem in this loop using an improved deep neural network [19].

Another difficulty in the complex networked industrial systems related to FDI is the sparsity of sensors. Because of financial reasons, it is rare to install a multitude of sensors along the connections of the interconnected subsystems in the network. Commonly, they are placed at the end of a connection or at some of the subsystem’s outputs. This motivates the development of an FDI method that groups network elements for fault identification [20,21].

As mentioned before, the interconnections that define the network topology make a networked dynamic system complex even if one considers simple elements (subsystems) in the network. Therefore, a practically important subclass of process systems is considered in this work where the elements are linear process systems connected by linear (possibly dynamic) connections.

Despite being a special case, it is a very important subclass because it can describe the dynamic behaviour of processes that serve our daily basic needs, e.g., domestic heating/cooling systems. Even some cellular processes belong to this subclass [22]. The fault that occurred in one of the subsystems is considered to be generated by an external source that can be treated as an extra input in a subsystem. Physically, this kind of fault represents some leakage phenomenon common inside networked linear process systems [23].

A model-based approach is proposed in this research to handle the fault diagnosis problem in a network of linear process systems, which may contain cycles/loops. The proposed approach does not need high computational costs. The burden of high computational power is common for fault diagnosis in a network [24].

The research questions that we are going to tackle are:

1. In a process network with loops, how can a fault be identified in a subsystem or group of subsystems regardless of the fault effect propagation through the network loops and branches?
2. Which sensor measurements are necessary to isolate a fault in a subsystem of a process network (or group of subsystems), and how can measurement noise be handled during fault diagnosis?
3. If fault isolation can be conducted, how can the magnitude of the fault be estimated?

In concordance with the addressed research questions, the main contributions of our proposed approach can be summarized as follows:

1. A fault diagnosis-oriented modelling approach is developed for such process networks in which the transport mechanisms can be described by stable and positive linear dynamic systems. The model includes the sources of the fault and indicates the sensor measurements. Based on this model, an analysis of how the fault input affects the steady states of the subsystems in the network is presented.
2. A model-based fault isolation approach is proposed for networks of linear process systems. The main benefit of this approach compared to previously reported ones is that, under reasonable assumptions, it can isolate the faults in a subsystem or group of subsystems regardless of the loops and ramifications in the network. The proposed algorithm also indicates which sensor measurements are necessary to perform the fault isolation tasks. Moreover, it is also applicable in the presence of measurement noise.
3. Finally, to determine the magnitude of the localized fault, the design of a disturbance observer-based asymptotic fault estimator is proposed.

## 2. Networked Linear Process Systems

Linear process systems are systems where there are only linear source terms, such as chemical reactions and phase changes (evaporation, condensation etc.), present besides the usual transport terms that include convection, diffusion and transfer through phase boundaries [22]. Although this is a special case, linear process systems are of great practical importance, e.g., heat exchanger networks and domestic heating/cooling systems.

Most linear process systems have a complex structure. They comprise linear subsystems connected by a linear network that can provide static or dynamic connections between the subsystems. Such composite linear process systems are called networked linear process systems. This section describes the dynamic modelling of networked linear process systems where we consider that there are j=1…N subsystems.

### 2.1. Linear Process Subsystems

For fault isolation and estimation, one usually applies simplified dynamic models where the effect of the considered fault(s) is also described. These dynamic models are constructed from first engineering principles (see [22]) where the state equations originate from dynamic balance equations for the conserved extensive quantities (such as energy, overall mass, component masses) in a balanced volume. However, they will be transformed to their intensive variable forms using algebraic constitutive equations. Therefore, the state variables in linear process subsystems are considered to be intensive variables, e.g., temperature, concentration, etc.

Intensive variables usually spatially vary within a balanced volume so that the conservation balance equations related to them are in the form of partial differential equations (PDEs). To obtain simple dynamic models suitable for fault isolation and estimation, the lumped form of process models is used to approximate PDEs to bring them into a set of ordinary differential equations (ODEs) [22].

It is important to note that the dynamic linear interconnections in networked process systems can be realized by PDEs to model linear process subsystems where the lumping produces a finite-dimensional model representing the distributed delay phenomenon in process systems [25].

#### 2.1.1. Modelling Assumptions

The following modelling assumptions are used in this paper:

A1 Only the transport of a single conserved extensive quantity (such as component mass, or energy) is considered in the process systems. Thus, we have either energy-transport or mass-transport systems. Heat exchanger networks and domestic heating/cooling systems belong to the linear energy-transport class. More details can be found in [26,27].
A2 Only linear convection and transfer is considered without any linear source.
A3 Constant overall mass and constant physicochemical parameters (such as density, specific heat, heat transfer coefficient, and convective flow rate) are assumed.
A4 One inlet and one outlet flow are considered where the inputs of the systems are the intensive variable (temperature or concentration) at the inlet and that of the balance volume with which transfer is assumed. Meanwhile, the output is the intensive variable (temperature or concentration) at the outlet.

#### 2.1.2. Model Equations

Based on the above general assumptions and considering spatially homogeneous lumping (i.e., the same parameters in every lump), the dynamic model equations of a linear process subsystem becomes a two input–single output (2ISO) LTI state-space model which has the form:

(1) $$\begin{array}{c} \begin{array}{cc} S^{\left(j\right)}:= & \left\{\begin{array}{c} {\dot{x}}^{\left(j\right)}=A^{\left(j\right)}x^{\left(j\right)}+B^{\left(j\right)}u_{X}^{\left(j\right)}\\ y^{\left(j\right)}=Cx^{\left(j\right)} \end{array}\right.\\ A^{\left(j\right)}= & \left[\begin{array}{cccccc} -(v^{\left(j\right)}+k_{E}^{\left(j\right)}) & 0 & 0 & \ldots & \ldots & 0\\ v^{\left(j\right)} & -(v^{\left(j\right)}+k_{E}^{\left(j\right)}) & 0 & \ldots & \ldots & 0\\ 0 & v^{\left(j\right)} & -(v^{\left(j\right)}+k_{E}^{\left(j\right)}) & \ldots & \ldots & 0\\ 0 & \ldots & \ldots & \ldots & \ldots & 0\\ 0 & \ldots & \ldots & \ldots & v^{\left(j\right)} & -(v^{\left(j\right)}+k_{E}^{\left(j\right)}) \end{array}\right]\\ B^{\left(j\right)}= & {\left[\begin{array}{cccc} v^{\left(j\right)} & 0 & \ldots & 0\\ k_{E}^{\left(j\right)} & k_{E}^{\left(j\right)} & \ldots & k_{E}^{\left(j\right)} \end{array}\right]}^{T},\;\;C=\left[\begin{array}{ccccc} 0 & 0 & 0 & \ldots & 1 \end{array}\right] \end{array} \end{array}$$

where $S^{\left(j\right)}$ is the general model of the *j*th subsystem with $x^{\left(j\right)}$ being the state variable, $y^{\left(j\right)}$ is the output variable, and $u_{X}^{\left(j\right)}={\left[u^{\left(j\right)}\;u_{E}^{\left(j\right)}\right]}^{T}$ is the input variable which consists of $u^{\left(j\right)}$ as the intensive variable of the *j*th subsystem at the inlet and $u_{E}^{\left(j\right)}$ as the intensive variable of the external balance volume (environment). Meanwhile, $v^{\left(j\right)}>0$ is the mass flow rate and $k_{E}^{\left(j\right)}>0$ is the transfer coefficient.

By looking at Equation (1), we can derive some important points:

1. As long as $k_{E}^{\left(j\right)}$ is positive, the model will always be stable because the eigenvalues are already stable shown by the negative sign of the diagonal entries of the $A^{\left(j\right)}$ matrix (see Gershgorin circle theorem for details [28]).
2. The $A^{\left(j\right)}\in R^{n\times n}$ matrix is a Metzler matrix by which all of its off-diagonal elements are non-negative. Thus, the matrix represents the time delayed differential equations and positive linear dynamical systems [29]. This is understandable because between the input and output states of the intensive variables in the subsystem, a propagation inside the transport element will occur contributing to the increase in delayed processes. This also implies the stability of the model because this matrix is sign stable and Hurwitz [30].
3. It is jointly controllable and observable because it is derived from conservation balance equations (it is a compartmental system) [31,32].

### 2.2. Interconnections and Topology of the Network

In the following, we consider process networks consisting of linear process subsystems described in Section 2.1 that are connected by static interconnections. In the case of dynamic interconnections, a special linear subsystem is used to represent the dynamics (i.e., the distributed delay) of the connection.

#### 2.2.1. Physically Meaningful Connections

Considering the modelling assumptions and the model equations of the applied linear process subsystem models, we can make physically meaningful connections between two subsystems (the *j*th and *ℓ*th, for example) by connecting part of the output flow of one (the *j*th) to the inlet flow of the other (the *ℓ*th). This implies that the characterizing intensive variable $y^{\left(j\right)}$ will determine the intensive variable $u^{\left(\ell \right)}$ and the inlet of the *ℓ*th subsystem.

#### 2.2.2. Equations Describing the Connections

In a realistic network, it is common to have a branching phenomenon in the interconnection between subsystems. Here, the Kirchoff law applies to the flows of extensive variables entering and exiting this interconnection. Considering the *ℓ*th junction, the sum of either the overall mass, component mass, or energy flows entering the junction ($v^{\left(\ell \right)}$) is equal to the sum of flows exiting ($v_{OUT}^{\left(\ell \right)}$), as shown in the following equation for the case of overall mass flows:

(2) $$\sum_{k\in IN^{\left(\ell \right)}}v^{\left(k\right)}=\sum_{l\in OUT^{\left(\ell \right)}}v^{\left(l\right)}=V^{\left(\ell \right)}$$

where *k* runs over the branches by which the flow enters the interconnection. Meanwhile, *l* runs over those where the flows exits the interconnection. $IN^{\left(\ell \right)}$ is the input set of the interconnection junction point, $OUT^{\left(\ell \right)}$ is the output set of the interconnection junction point, and *v* is measured in $\left[\frac{k𝘨}{s}\right]$.

Similar conservation equations apply for the energy flows $\dot{Q}$ in energy-transport systems or the component mass $m_{X}$ in mass-transport systems. However, to obtain the relations between the intensive variables (temperature *T* or concentration $c_{X}$, respectively) among the subsystems in this networked process systems, we substitute the algebraic equations representing the relationship of the intensive–extensive variables into the mass conservation results. These algebraic relationships are in the following general form:

(3) $$Q=Mc_{P}T,\;\;m_{X}=Mc_{X}$$

where *M* is the overall mass and $c_{P}$ is the specific heat in the balance volume.

Then, we can substitute relations in (3) into the Kirchhoff law in Equation (2) while taking into account that the value of the intensive variable for all the outflows is the same. This way, one obtains the linear algebraic equations for the intensive variables *T* or $c_{X}$ at the *ℓ*th junction in the following general form:

(4) $$\begin{array}{c} \begin{array}{ccc} & & \sum_{k\in IN^{\left(\ell \right)}}\frac{v^{\left(k\right)}}{V^{\left(\ell \right)}}T^{\left(k\right)}=T^{\left(l\right)}\;\;\mathrm{or}\;\;\sum_{k\in IN^{\left(\ell \right)}}\frac{v^{\left(k\right)}}{V^{\left(\ell \right)}}c_{X}^{\left(k\right)}=c_{X}^{\left(l\right)}\\ & & \sum_{k\in IN^{\left(\ell \right)}}\frac{v^{\left(k\right)}}{V^{\left(\ell \right)}}y^{\left(k\right)}=u^{\left(l\right)}\;\;,\;\;\forall \;l\in OUT^{\left(\ell \right)} \end{array} \end{array}$$

#### 2.2.3. Network Topology

An underlying graph (G) can be associated with the process network. The edges of the graph represent the dynamic subsystems of the network. Meanwhile, the vertices represent the interconnections among the subsystems which have no dynamics.

**Figure 1:**
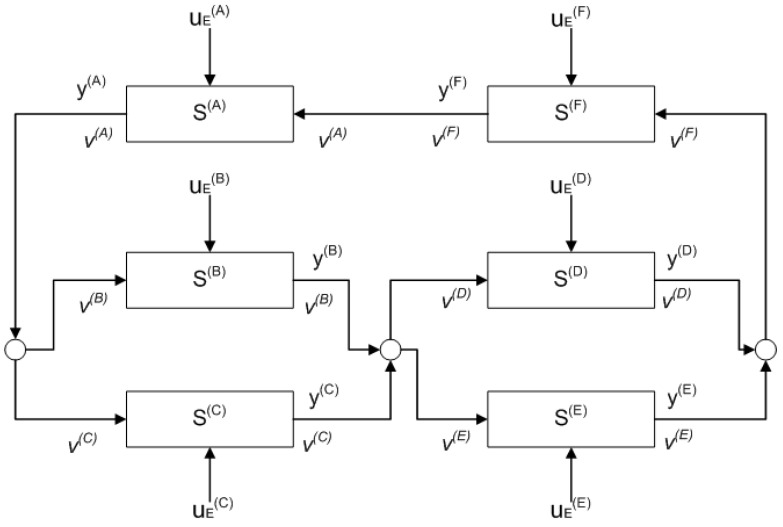
Example of a process network.

**Example 1.**  *Consider a network composed of N subsystems. Figure 1 gives an example for this network of transport elements where* N=6 *and* $v^{\left(j\right)}$ *is the mass flow rate of the jth subsystem.*

*Based on Equation *(2)*, we can derive some relations between* $v^{\left(j\right)}$ *in Figure 1 as follows:* 

(5) $$\begin{array}{c} \begin{array}{cc} 1.\;\; & v^{\left(A\right)}=v^{\left(B\right)}+v^{\left(C\right)}\\ 2.\;\; & v^{\left(B\right)}+v^{\left(C\right)}=v^{\left(D\right)}+v^{\left(E\right)}=V^{\left(1\right)}\\ 3.\;\; & v^{\left(D\right)}+v^{\left(E\right)}=v^{\left(F\right)}\\ 4.\;\; & v^{\left(F\right)}=v^{\left(A\right)} \end{array} \end{array}$$

*If we want to obtain some relations in terms of temperature (either* $u^{\left(j\right)}$ *or* $y^{\left(j\right)}$*) among the subsystems from the same Figure 1, we can substitute Equation *(3)* into each relation in Equation *(5)*. Thus, we obtain:* 

(6) $$\begin{array}{c} \begin{array}{cc} 1.\;\; & {\dot{Q}}^{\left(A\right)}={\dot{Q}}^{\left(B\right)}+{\dot{Q}}^{\left(C\right)},\;so\;that\;y^{\left(A\right)}=u^{\left(B\right)}=u^{\left(C\right)}\\ 2.\;\; & {\dot{Q}}^{\left(B\right)}+{\dot{Q}}^{\left(C\right)}={\dot{Q}}^{\left(D\right)}+{\dot{Q}}^{\left(E\right)},\;so\;that\;\frac{v^{\left(B\right)}}{V^{\left(1\right)}}y^{\left(B\right)}+\frac{v^{\left(C\right)}}{V^{\left(1\right)}}y^{\left(C\right)}=u^{\left(D\right)}=u^{\left(E\right)}\\ 3.\;\; & {\dot{Q}}^{\left(D\right)}+{\dot{Q}}^{\left(E\right)}={\dot{Q}}^{\left(F\right)},\;so\;that\;\frac{v^{\left(D\right)}}{v^{\left(F\right)}}y^{\left(D\right)}+\frac{v^{\left(E\right)}}{v^{\left(F\right)}}y^{\left(E\right)}=u^{\left(F\right)}\\ 4.\;\; & {\dot{Q}}^{\left(F\right)}={\dot{Q}}^{\left(A\right)},\;so\;that\;y^{\left(F\right)}=u^{\left(A\right)} \end{array} \end{array}$$

*Note that in the considered process network model, all the edges are part of at least one loop in the graph.*

## 3. Faults in the Network

### 3.1. Fault Modelling

In this work, the considered fault is a constant input signal which additively modifies the external intensive variable input signal $u_{E}^{\left(j\right)}$. The faulty external variable input signal $u_{Ef}^{\left(j\right)}$ has the form:

(7) $$u_{Ef}^{\left(j\right)}=u_{E}^{\left(j\right)}+f^{\left(j\right)}$$

where $f^{\left(j\right)}$ is the fault signal in the *j*th subsystem.

Such fault modelling can describe several fault events: the unforeseen appearance of an unknown external source, or change in the heat transfer coefficient $k_{E}^{\left(j\right)}$.

If there is a change $f_{k}^{\left(j\right)}$ in the heat transfer coefficient, i.e., $k_{Ef}^{\left(j\right)}=k_{E}^{\left(j\right)}+f_{k}^{\left(j\right)}$, then the second input of the model (1) can be rephrased as:

(8) $$\begin{array}{c} \begin{array}{cc} \left(k_{E}^{\left(j\right)}+f_{k}^{\left(j\right)}\right)u_{E}^{\left(j\right)} & =k_{E}^{\left(j\right)}\left(u_{E}^{\left(j\right)}+f^{\left(j\right)}\right),\\ \mathrm{where}\;\;f^{\left(j\right)} & =\frac{f_{k}^{\left(j\right)}u_{E}^{\left(j\right)}}{k_{E}^{\left(j\right)}}. \end{array} \end{array}$$

During the fault diagnosis algorithm design, we assumed that:

A5 The probability of multiple fault events happening at the same time in the network is negligible (weak fault isolation), i.e., there are no simultaneous faults during the fault isolation and estimation processes.

### 3.2. The Fault’s Effect in Different Measurement Positions in the Network

First, to investigate the fault’s effect on the subsystems of the network, the state space model in Equation (1) is converted into input–output realization as follows:

(9) $$\begin{array}{c} \begin{array}{cc} y^{\left(j\right)}\left(s\right) & =S_{1}^{\left(j\right)}\left(s\right)u^{\left(j\right)}\left(s\right)+S_{2}^{\left(j\right)}\left(s\right)u_{E}^{\left(j\right)}\left(s\right)\\ S_{1}^{\left(j\right)}\left(s\right) & ={\left(\frac{v^{\left(j\right)}}{s+v^{\left(j\right)}+k_{E}^{\left(j\right)}}\right)}^{n}\\ S_{2}^{\left(j\right)}\left(s\right) & =\sum_{h=1}^{n}\left[\frac{{\left(v^{\left(j\right)}\right)}^{\left(h-1\right)}}{{\left(s+v^{\left(j\right)}+k_{E}^{\left(j\right)}\right)}^{h}}\right]k_{E}^{\left(j\right)} \end{array} \end{array}$$

where $S_{1}^{\left(j\right)}\left(s\right)$ is the transfer function in Laplace domain from $u^{\left(j\right)}\left(s\right)$ to $y^{\left(j\right)}\left(s\right)$ and $S_{2}^{\left(j\right)}\left(s\right)$ is the transfer function in Laplace domain from $u_{E}^{\left(j\right)}\left(s\right)$ to $y^{\left(j\right)}\left(s\right)$. Zero initial states are assumed.

**Figure 2:**
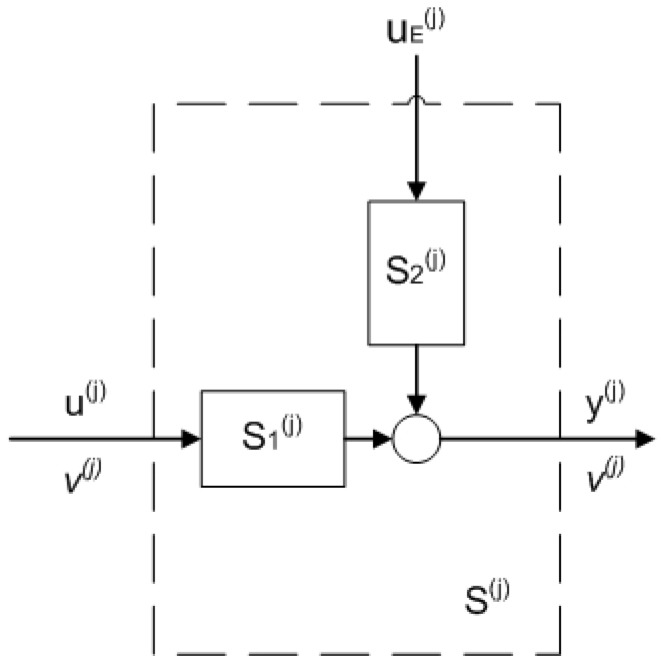
Input–output representation of a subsystem.

Figure 2 shows the proposed realization for the fault effect analysis. For the sake of convenience, the notation $S_{i}^{\left(j\right)}$ will be used instead of $S_{i}^{\left(j\right)}\left(s\right)$ from here on.

**Figure 3:**
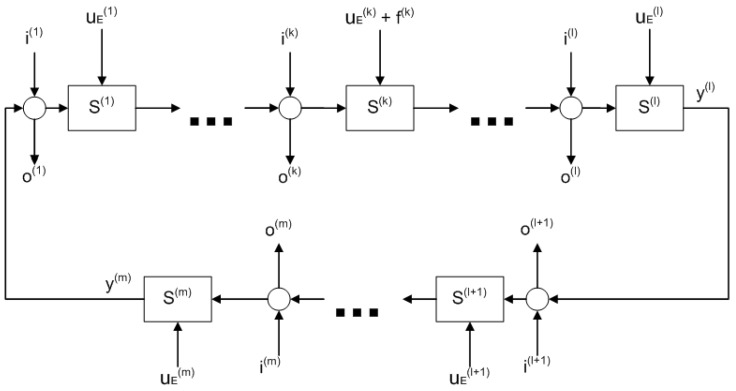
Diagram of a loop/cycle with fault in the process network.

Now, consider a loop in the process network as shown in Figure 3. There, $S^{\left(j\right)}$ represents the block diagram of the *j*th subsystem which contains $S_{1}^{\left(j\right)}$ and $S_{2}^{\left(j\right)}$ as shown in Figure 2. Meanwhile, $i^{\left(j\right)}$, where j=1…m, represents the inflows of the subsystems that are not part of the loop (possible joining connections), and $o^{\left(j\right)}$, where j=1…m, represents the outflows from the loop in splitting connections.

It is considered that the sensors are placed at the outputs $y^{\left(l\right)}$ and $y^{\left(m\right)}$ where m≥2 and 0<l<m.

The fault $f^{\left(k\right)}$ is represented by a constant input in the *k*th subsystem, where 0<k<l, which enters the subsystem from the same channel as the external source $u_{E}^{\left(k\right)}$.

For fault effect analysis, the final value theorem (FVT) of the Laplace transform is applied:

(10) $$\lim_{t\to \infty }f\left(t\right)=\lim_{s\to 0}sF\left(s\right)$$

where F(s) is the Laplace transform of f(t).

To calculate the steady state value of the *l*th subsystem’s output in the fault-free case $f^{\left(k\right)}=0$ (see Figure 2), the FVT is applied to the previous transfer functions along with the superposition principle. By assuming that the inputs are step functions with zero initial conditions, it yields:

(11) $$\begin{array}{c} \begin{array}{cc} y_{ss}^{\left(l\right)} & =\sum_{j=1}^{l}\left[\frac{{∥S_{2}^{\left(j\right)}∥}_{0}\prod_{h=j+1}^{l}{∥S_{1}^{\left(h\right)}∥}_{0}}{1-\prod_{h=1}^{m}{∥S_{1}^{\left(h\right)}∥}_{0}}\right]\left(\frac{v^{\left(j\right)}}{V^{\left(j\right)}}\right)u_{E}^{\left(j\right)}\\ & +\sum_{j=l+1}^{m}\left[\frac{{∥S_{2}^{\left(j\right)}∥}_{0}\prod_{h=j+1}^{m}∥S_{1}^{\left(h\right)}{∥}_{0}\prod_{h=1}^{l}{∥S_{1}^{\left(h\right)}∥}_{0}}{1-\prod_{h=1}^{m}{∥S_{1}^{\left(h\right)}∥}_{0}}\right]\left(\frac{v^{\left(j\right)}}{V^{\left(j\right)}}\right)u_{E}^{\left(j\right)}\\ & +\sum_{j=1}^{l}\left[\frac{\prod_{h=j}^{m}{∥S_{1}^{\left(h\right)}∥}_{0}}{1-\prod_{h=1}^{m}{∥S_{1}^{\left(h\right)}∥}_{0}}\right]\left(\frac{v^{\left(j\right)}}{V^{\left(j\right)}}\right)i_{ss}^{\left(j\right)}+\sum_{j=l+1}^{m}\left[\frac{\prod_{h=j}^{m}∥S_{1}^{\left(h\right)}{∥}_{0}\prod_{h=1}^{l}{∥S_{1}^{\left(h\right)}∥}_{0}}{1-\prod_{h=1}^{m}{∥S_{1}^{\left(h\right)}∥}_{0}}\right]\left(\frac{v^{\left(j\right)}}{V^{\left(j\right)}}\right)i_{ss}^{\left(j\right)} \end{array} \end{array}$$

where $V^{\left(j\right)}$ is the sum of the mass flow rate passing through the *j*th joining/splitting connection before the *j*th subsystem input, $y_{ss}^{\left(l\right)}$ is the steady state value of $y^{\left(l\right)}$ when there is no fault, $i_{ss}^{\left(j\right)}$ is the steady state value of $i^{\left(j\right)}$, and ${∥\cdot ∥}_{0}$ is the steady state gain of the related transfer function. $u_{E}^{\left(j\right)}$ is assumed to be constant.

Since the addressed subsystem class is positive (see Section 2.1), the steady state gains are also always positive.

Note that the terms of $\left(1-\prod ∥S_{1}^{\left(j\right)}{∥}_{0}\right)$ appear because of the loop. Meanwhile, the terms of $\left(\frac{v^{\left(j\right)}}{V^{\left(j\right)}}\right)$ come from the mass/energy conservation balance (see Section 2.2).

When a step-like fault arises in the *k*th subsystem ($f^{\left(k\right)}\neq 0$), we obtain:

(12) $$\begin{array}{c} \begin{array}{cc} y_{fss}^{\left(l\right)} & =\left[\frac{{∥S_{2}^{\left(k\right)}∥}_{0}\prod_{j=k+1}^{l}{∥S_{1}^{\left(j\right)}∥}_{0}}{1-\prod_{j=1}^{m}{∥S_{1}^{\left(j\right)}∥}_{0}}\right]\left(\frac{v^{\left(k\right)}}{V^{\left(k\right)}}\right)f^{\left(k\right)}\\ & +\sum_{j=1}^{l}\left[\frac{{∥S_{2}^{\left(j\right)}∥}_{0}\prod_{h=j+1}^{l}{∥S_{1}^{\left(h\right)}∥}_{0}}{1-\prod_{h=1}^{m}{∥S_{1}^{\left(h\right)}∥}_{0}}\right]\left(\frac{v^{\left(j\right)}}{V^{\left(j\right)}}\right)u_{E}^{\left(j\right)}\\ & +\sum_{j=l+1}^{m}\left[\frac{{∥S_{2}^{\left(j\right)}∥}_{0}\prod_{h=j+1}^{m}∥S_{1}^{\left(h\right)}{∥}_{0}\prod_{h=1}^{l}{∥S_{1}^{\left(h\right)}∥}_{0}}{1-\prod_{h=1}^{m}{∥S_{1}^{\left(h\right)}∥}_{0}}\right]\left(\frac{v^{\left(j\right)}}{V^{\left(j\right)}}\right)u_{E}^{\left(j\right)}\\ & +\sum_{j=1}^{l}\left[\frac{\prod_{h=j}^{m}{∥S_{1}^{\left(h\right)}∥}_{0}}{1-\prod_{h=1}^{m}{∥S_{1}^{\left(h\right)}∥}_{0}}\right]\left(\frac{v^{\left(j\right)}}{V^{\left(j\right)}}\right)i_{ss}^{\left(j\right)}+\sum_{j=l+1}^{m}\left[\frac{\prod_{h=j}^{m}∥S_{1}^{\left(h\right)}{∥}_{0}\prod_{h=1}^{l}{∥S_{1}^{\left(h\right)}∥}_{0}}{1-\prod_{h=1}^{m}{∥S_{1}^{\left(h\right)}∥}_{0}}\right]\left(\frac{v^{\left(j\right)}}{V^{\left(j\right)}}\right)i_{ss}^{\left(j\right)} \end{array} \end{array}$$

where $y_{fss}^{\left(j\right)}$ is the steady state value of $y^{\left(j\right)}$ in the presence of a fault.

Now, by subtracting Equation (11) from Equation (12), we obtain the deviation of the faulty output related to the fault-free case:

(13) $$\begin{array}{c} \begin{array}{cc} y_{fss}^{\left(l\right)}-y_{ss}^{\left(l\right)} & =\left[\frac{{∥S_{2}^{\left(k\right)}∥}_{0}\prod_{j=k+1}^{l}{∥S_{1}^{\left(j\right)}∥}_{0}}{1-\prod_{j=1}^{m}{∥S_{1}^{\left(j\right)}∥}_{0}}\right]\left(\frac{v^{\left(k\right)}}{V^{\left(k\right)}}\right)f^{\left(k\right)} \end{array} \end{array}$$

The difference between the faulty and fault-free outputs of the *m*th subsystem can be computed similarly:

(14) $$\begin{array}{c} \begin{array}{cc} y_{fss}^{\left(m\right)}-y_{ss}^{\left(m\right)} & =\left[\frac{{∥S_{2}^{\left(k\right)}∥}_{0}\prod_{j=k+1}^{m}{∥S_{1}^{\left(j\right)}∥}_{0}}{1-\prod_{j=1}^{m}{∥S_{1}^{\left(j\right)}∥}_{0}}\right]\left(\frac{v^{\left(k\right)}}{V^{\left(k\right)}}\right)f^{\left(k\right)} \end{array} \end{array}$$

Equations (13) and (14) show that the fault influences all subsystems in the loop. However, the fault effect on the outputs of the subsystems is calculable.

**Figure 4:**
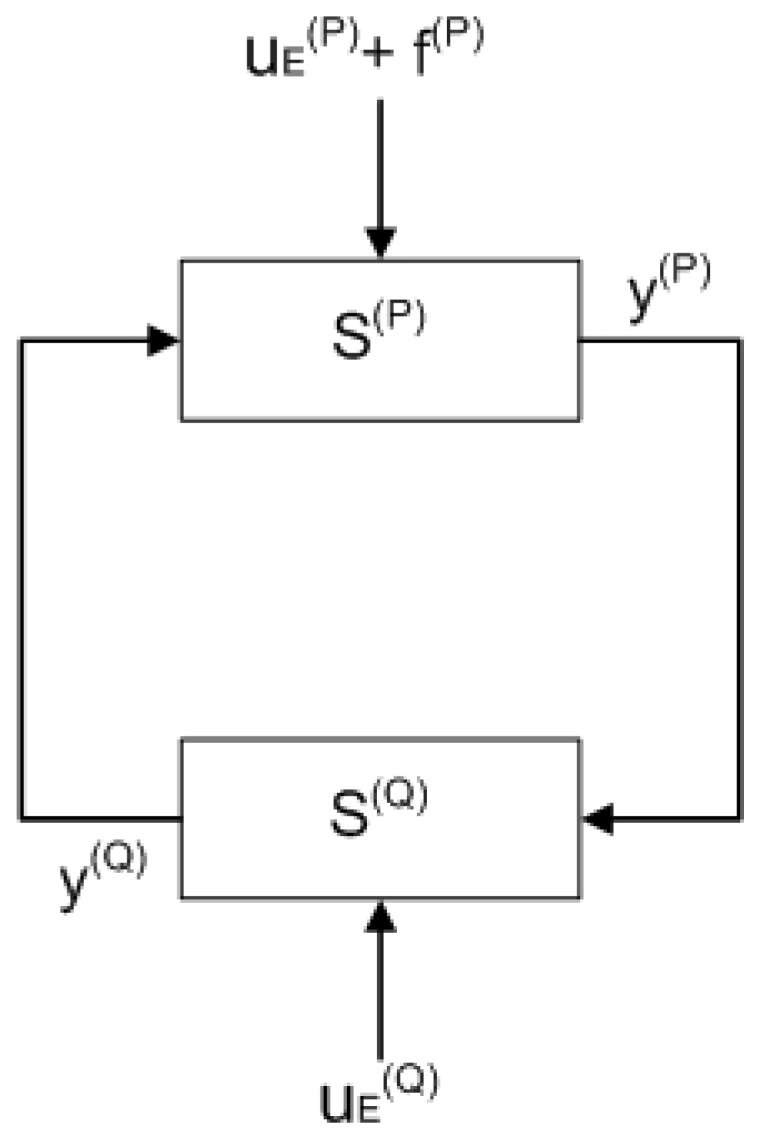
Diagram of two subsystems in a loop with a fault.

**Example 2.**  *Figure 4 presents two connected subsystems which form a loop. The input–output models of the subsystems are* $S^{\left(P\right)}$ *and* $S^{\left(Q\right)}$*, respectively, where each contains* $S_{1}^{\left(P\right)}$*,* $S_{2}^{\left(P\right)}$*,* $S_{1}^{\left(Q\right)}$*, and* $S_{2}^{\left(Q\right)}$ *as shown in Figure 2.*

*No joining and splitting connections are assumed (*$i^{\left(j\right)}=o^{\left(j\right)}=0$*), so* $v^{\left(P\right)}=v^{\left(Q\right)}=V$ *which leads to* $\frac{v^{\left(j\right)}}{V^{\left(j\right)}}=1$ *where* j=P,Q*. Moreover,* $y^{\left(P\right)}=u^{\left(Q\right)}$ *and* $y^{\left(Q\right)}=u^{\left(P\right)}$*.*

*From Equations *(13)* and *(14)*, the step-like fault* $f^{\left(P\right)}\neq 0$ *generates deviations in the subsystems as follows:* 

(15) $$\begin{array}{c} \begin{array}{cc} y_{fss}^{\left(P\right)}-y_{ss}^{\left(P\right)} & =\left(\frac{∥S_{2}^{\left(P\right)}{∥}_{0}}{1-∥S_{1}^{\left(P\right)}{∥}_{0}{∥S_{1}^{\left(Q\right)}∥}_{0}}\right)f^{\left(P\right)}\\ y_{fss}^{\left(Q\right)}-y_{ss}^{\left(Q\right)} & =\left(\frac{∥S_{2}^{\left(P\right)}{∥}_{0}{∥S_{1}^{\left(Q\right)}∥}_{0}}{1-∥S_{1}^{\left(P\right)}{∥}_{0}{∥S_{1}^{\left(Q\right)}∥}_{0}}\right)f^{\left(P\right)} \end{array} \end{array}$$

## 4. Fault Diagnosis

### 4.1. Problem Formulation

Given a process network as defined in Section 2.2, consider that a fault event arises in one of the subsystem in the network. As a consequence of the branches and loops in the underlying graph (G) of the process network, the effect of this fault could induce deviations in the outputs of all the subsystems. Moreover, due to the loops, the fault could propagate back to the input of the faulty subsystem as well. The loops and network branches make the fault effect propagation barely traceable.

The presence of a fault in the network can simply be detected by comparing the measured outputs of the subsystems in the network with the predicted outputs based on a reliable model of the corresponding subsystem. However, due to fault effect propagation, the localization of the fault source in the network is a difficult task.

Recall that the subsystems represent the edges in the underlying graph G of the process network. We consider a directed path of subsystems $\left\{S^{\left(1\right)}\ldots S^{\left(l\right)}\right\}$ where l>0 such that this path may be part of at least one simple loop of G.

Let a fault event happen in a subsystem in the network. Formulate the following fault diagnosis problems:

- Consider a path of *l* subsystems that can be part of a loop consisting of m>l subsystems (see Figure 3). We must determine whether the fault occurred in the addressed path. Furthermore, we must determine which measurements are necessary to perform the isolation problem (sensors placement) in this path.
- If the fault has been isolated in one subsystem, an estimation algorithm must be designed that outputs the magnitude of the fault.

Note that the path can be part of more than one loop in the graph of the process network, e.g., in Figure 1, the path $\left\{S^{\left(A\right)},\;S^{\left(F\right)}\right\}$ is in more than one simple loop. For an algorithm that finds all the simple loops in a graph, see, e.g., [33]. In this case, such a loop should be chosen that is more representative for the fault isolation process, e.g., from the perspective of the sensor placement.

The formulated diagnosis problem can specify whether the fault happened in a group of subsystems (or one subsystem for l=1), or in some other part of the network. However, the diagnosis process can be repeated for different groups (or subsystems). Thus, by exhaustive search, the fault in the network can be localized.

The fault isolation algorithm is designed by applying the fault effect analysis as presented in Section 3.2. For fault estimator design, we take the PI observer approach.

### 4.2. Fault Isolation

To derive a fault isolation algorithm, we consider that two sensors are placed in a loop at two different locations (see Figure 3). They measure the output of the *l*th and *m*th subsystems.

The isolation logic is based on Equations (13) and (14). By subtracting these equations, we obtain:

(16) $$\left(y_{fss}^{\left(l\right)}-y_{ss}^{\left(l\right)}\right)-\left(y_{fss}^{\left(m\right)}-y_{ss}^{\left(m\right)}\right)=\left[\left(\frac{{∥S_{2}^{\left(k\right)}∥}_{0}\prod_{j=k+1}^{l}{∥S_{1}^{\left(j\right)}∥}_{0}}{1-\prod_{j=1}^{m}{∥S_{1}^{\left(j\right)}∥}_{0}}\right)\left(1-\prod_{j=l+1}^{m}∥S_{1}^{\left(j\right)}{)∥}_{0}\right)\right]\left(\frac{v^{\left(k\right)}}{V^{\left(k\right)}}\right)f^{\left(k\right)}$$

We make the following assumptions on the steady state gains of the subsystems:

A6 Either every ${∥S_{1}^{\left(j\right)}∥}_{0}\in \left(0,1\right)$ or every ${∥S_{1}^{\left(j\right)}∥}_{0}>1$;
A7 ${∥S_{2}^{\left(j\right)}∥}_{0}>0\;\forall j$.

In the view of Equation (16), if these assumptions hold, then

(17) $$\left(\frac{{∥S_{2}^{\left(k\right)}∥}_{0}\prod_{j=k+1}^{l}{∥S_{1}^{\left(j\right)}∥}_{0}}{1-\prod_{j=1}^{m}{∥S_{1}^{\left(j\right)}∥}_{0}}\right)\left(1-\prod_{j=l+1}^{m}∥S_{1}^{\left(j\right)}{)∥}_{0}\right)>0$$

Note that A6 and A7 hold true for the subsystem’s model introduced in Section 2.1; if we apply the FVT to Equation (9), we obtain:

(18) $$\begin{array}{c} \begin{array}{cc} & {∥S_{1}^{\left(j\right)}∥}_{0}=\lim_{s\to 0}S_{1}^{\left(j\right)}\left(s\right)={\left(\frac{v^{\left(j\right)}}{v^{\left(j\right)}+k_{E}^{\left(j\right)}}\right)}^{n}\in \left(0,1\right)\\ & {∥S_{2}^{\left(j\right)}∥}_{0}=\lim_{s\to 0}S_{2}^{\left(j\right)}\left(s\right)=\sum_{h=1}^{n}\left[\frac{{\left(v^{\left(j\right)}\right)}^{\left(h-1\right)}}{{\left(v^{\left(j\right)}+k_{E}^{\left(j\right)}\right)}^{h}}\right]k_{E}^{\left(j\right)}>0 \end{array} \end{array}$$

Furthermore, by assumptions A6 and A7, Equation (16) leads to:

(19) $$\begin{array}{c} \begin{array}{cc} \left(y_{fss}^{\left(l\right)}-y_{ss}^{\left(l\right)}\right)>\left(y_{fss}^{\left(m\right)}-y_{ss}^{\left(m\right)}\right) & \;for\;f^{\left(k\right)}>0\;and\\ \left(y_{fss}^{\left(l\right)}-y_{ss}^{\left(l\right)}\right)<\left(y_{fss}^{\left(m\right)}-y_{ss}^{\left(m\right)}\right) & \;for\;f^{\left(k\right)}<0\\ ∴∥y_{fss}^{\left(l\right)}-y_{ss}^{\left(l\right)}∥-∥y_{fss}^{\left(m\right)}-y_{ss}^{\left(m\right)}∥>0 & \;for\;f^{\left(k\right)}\neq 0 \end{array} \end{array}$$

where ∥·∥ is the absolute value of the related function.

Thus, Equation (19) shows that $∥y_{fss}^{\left(l\right)}-y_{ss}^{\left(l\right)}∥-∥y_{fss}^{\left(m\right)}-y_{ss}^{\left(m\right)}∥>0$ when a fault occurs in a subsystem between $S^{\left(1\right)}$ and $S^{\left(l\right)}$.

With the same assumptions, we can perform the same derivation to obtain $∥y_{fss}^{\left(l\right)}-y_{ss}^{\left(l\right)}∥-∥y_{fss}^{\left(m\right)}-y_{ss}^{\left(m\right)}∥<0$ when a fault occurs in a subsystem between $S^{\left(l+1\right)}$ and $S^{\left(m\right)}$.

For implementation of the fault isolation algorithm, the fault-free steady state value $y_{ss}^{\left(l\right)}$ and $y_{ss}^{\left(m\right)}$ have to be known prior, or they have to be computed. In view of the relation in (11), to compute $y_{ss}^{\left(l\right)}$ and $y_{ss}^{\left(m\right)}$, the steady state value of the inputs $i_{ss}^{\left(j\right)}$ and $u_{E}^{\left(j\right)}$ have to be measured.

To conclude, the fault isolation can be performed according to Algorithm 1 as follows:
**Algorithm 1** Fault isolation algorithm.Measure $y_{fss}^{\left(l\right)}$ and $y_{fss}^{\left(m\right)}$ in steady state.Compute $y_{ss}^{\left(l\right)}$ and $y_{ss}^{\left(m\right)}$.Isolate the fault:-If $∥y_{fss}^{\left(l\right)}-y_{ss}^{\left(l\right)}∥=∥y_{fss}^{\left(m\right)}-y_{ss}^{\left(m\right)}∥=0$, then no fault event occurred.-If $∥y_{fss}^{\left(l\right)}-y_{ss}^{\left(l\right)}∥-∥y_{fss}^{\left(m\right)}-y_{ss}^{\left(m\right)}∥>0$, then the fault occurred before *l* and after *m*.-If $∥y_{fss}^{\left(l\right)}-y_{ss}^{\left(l\right)}∥-∥y_{fss}^{\left(m\right)}-y_{ss}^{\left(m\right)}∥<0$, then the fault occurred before *m* and after *l*.

### 4.3. Fault Isolation in the Presence of Measurement Noise

In a realistic environment, it has to be considered that the measurements on the subsystems are affected by signal noise. In model (1), the noise inputs that influence the model’s inputs and outputs are introduced as:

(20) $$\begin{array}{c} \left\{\begin{array}{c} {\dot{x}}^{\left(j\right)}=A^{\left(j\right)}x^{\left(j\right)}+B^{\left(j\right)}\left(u_{X}^{\left(j\right)}+w_{uX}^{\left(j\right)}\right)\\ y^{\left(j\right)}=Cx^{\left(j\right)}+w_{y}^{\left(j\right)} \end{array}\right. \end{array}$$

where $w_{uX}^{\left(j\right)}={\left[w_{u}^{\left(j\right)}\;w_{E}^{\left(j\right)}\right]}^{T}$.

The following assumptions are considered for signal noise:

A8 ${∥w_{u}^{\left(j\right)}\left(t\right)∥}_{\infty }\leq w_{uM}^{\left(j\right)},\;∥w_{E}^{\left(j\right)}{\left(t\right)∥}_{\infty }\leq w_{EM}^{\left(j\right)},\;{∥w_{y}^{\left(j\right)}\left(t\right)∥}_{\infty }\leq w_{yM}^{\left(j\right)}$
  where ${∥\cdot ∥}_{\infty }$ is the infinity norm of the related function.
A9 $w_{u}^{\left(j\right)}\left(t\right),\;w_{E}^{\left(j\right)}\left(t\right),\;w_{y}^{\left(j\right)}\left(t\right)$ are locally integrable.
A10 The signal noise is unbiased with known variances:
  $w_{E}^{\left(j\right)}\left(t\right)∼\left(0,R_{u}^{\left(j\right)}\right),\;w_{y}^{\left(j\right)}\left(t\right)∼\left(0,Q_{y}^{\left(j\right)}\right)$.
A11 $w_{u}^{\left(j\right)}\left(t\right),\;w_{E}^{\left(j\right)}\left(t\right),\;w_{y}^{\left(j\right)}\left(t\right)$ are mutually uncorrelated with both each other and system states.

The input–output model in (9) in the presence of newly considered signal noise takes the form:

(21) $$\begin{array}{c} \begin{array}{cc} y_{n}^{\left(j\right)}\left(s\right) & =S_{1}^{\left(j\right)}\left(s\right)\left[u^{\left(j\right)}\left(s\right)+w_{u}^{\left(j\right)}\left(s\right)\right]+S_{2}^{\left(j\right)}\left(s\right)\left[u_{E}^{\left(j\right)}\left(s\right)+w_{E}^{\left(j\right)}\left(s\right)\right]+w_{y}^{\left(j\right)}\left(s\right)\\ y_{n}^{\left(j\right)}\left(s\right) & =y^{\left(j\right)}\left(s\right)+\left[S_{1}^{\left(j\right)}\left(s\right)w_{u}^{\left(j\right)}\left(s\right)+S_{2}^{\left(j\right)}\left(s\right)w_{E}^{\left(j\right)}\left(s\right)+w_{y}^{\left(j\right)}\left(s\right)\right]\\ y_{n}^{\left(j\right)}\left(s\right) & =y^{\left(j\right)}\left(s\right)+w^{\left(j\right)}\left(s\right). \end{array} \end{array}$$

where $y_{n}^{\left(j\right)}$ is the subsystem’s output in the presence of noise and $w^{\left(j\right)}$ is the noise’s effect where

(22) $${∥w^{\left(j\right)}\left(t\right)∥}_{\infty }\leq w_{M}^{\left(j\right)}.$$

As the noise from each subsystem propagates inside the loop, the measurement at both points *l* and *m* in the network are affected. Thus, to compensate for noise in the fault isolation process, a suitable threshold needs to be specified.

By applying the FVT and superposition principle, the noise’s effect on the measurements at sensors *l* and *m* can be computed, obtaining:

(23) $$\begin{array}{c} \begin{array}{cc} y_{fnss}^{\left(l\right)} & =y_{fss}^{\left(l\right)}+\sum_{j=1}^{l}\left[\frac{\prod_{h=j+1}^{l}{∥S_{1}^{\left(h\right)}∥}_{0}}{1-\prod_{h=1}^{m}{∥S_{1}^{\left(h\right)}∥}_{0}}\right]\left(\frac{v^{\left(j\right)}}{V^{\left(j\right)}}\right)w^{\left(j\right)}+\sum_{j=l+1}^{m}\left[\frac{\prod_{h=j+1}^{m}∥S_{1}^{\left(h\right)}{∥}_{0}\prod_{h=1}^{l}{∥S_{1}^{\left(h\right)}∥}_{0}}{1-\prod_{h=1}^{m}{∥S_{1}^{\left(h\right)}∥}_{0}}\right]\left(\frac{v^{\left(j\right)}}{V^{\left(j\right)}}\right)w^{\left(j\right)}\\ y_{fnss}^{\left(m\right)} & =y_{fss}^{\left(m\right)}+\sum_{j=1}^{m-1}\left[\frac{\prod_{h=j+1}^{m}{∥S_{1}^{\left(h\right)}∥}_{0}}{1-\prod_{h=1}^{m}{∥S_{1}^{\left(h\right)}∥}_{0}}\right]\left(\frac{v^{\left(j\right)}}{V^{\left(j\right)}}\right)w^{\left(j\right)}+\left[\frac{\prod_{h=1}^{m}{∥S_{1}^{\left(h\right)}∥}_{0}}{1-\prod_{h=1}^{m}{∥S_{1}^{\left(h\right)}∥}_{0}}\right]\left(\frac{v^{\left(m\right)}}{V^{\left(m\right)}}\right)w^{\left(m\right)} \end{array} \end{array}$$

where $y_{fnss}^{\left(j\right)}$ is the steady state value of $y^{\left(j\right)}$ in the presence of a fault and noise, $w^{\left(l\right)}$ is the noise’s effect on the measurement at sensor *l*, and $w^{\left(m\right)}$ is the noise’s effect on the measurement at sensor *m*.

With the addition of the noise’s effect, Equation (16) becomes:

(24) $$\begin{array}{c} \begin{array}{cc} \left(y_{fnss}^{\left(l\right)}-y_{ss}^{\left(l\right)}\right)-\left(y_{fnss}^{\left(m\right)}-y_{ss}^{\left(m\right)}\right) & =\left[\left(\frac{{∥S_{2}^{\left(k\right)}∥}_{0}\prod_{j=k+1}^{l}{∥S_{1}^{\left(j\right)}∥}_{0}}{1-\prod_{j=1}^{m}{∥S_{1}^{\left(j\right)}∥}_{0}}\right)\left(1-\prod_{j=l+1}^{m}∥S_{1}^{\left(j\right)}{)∥}_{0}\right)\right]\left(\frac{v^{\left(k\right)}}{V^{\left(k\right)}}\right)f^{\left(k\right)}\\ & +\sum_{j=1}^{l}\underset{S_{wl}^{\left(j\right)}}{\underset{︸}{\left[\frac{\prod_{h=j+1}^{l}{∥S_{1}^{\left(h\right)}∥}_{0}\left(1-\prod_{h=l+1}^{m}{∥S_{1}^{\left(h\right)}∥}_{0}\right)}{1-\prod_{h=1}^{m}{∥S_{1}^{\left(h\right)}∥}_{0}}\right]\left(\frac{v^{\left(j\right)}}{V^{\left(j\right)}}\right)}}w^{\left(j\right)}\\ & +\sum_{j=l+1}^{m}\underset{S_{wm}^{\left(j\right)}}{\underset{︸}{\left[\frac{\prod_{h=j+1}^{m}{∥S_{1}^{\left(h\right)}∥}_{0}\left(\prod_{h=1}^{l}{∥S_{1}^{\left(h\right)}∥}_{0}-1\right)}{1-\prod_{h=1}^{m}{∥S_{1}^{\left(h\right)}∥}_{0}}\right]\left(\frac{v^{\left(j\right)}}{V^{\left(j\right)}}\right)}}w^{\left(j\right)} \end{array} \end{array}$$

Now, for measurements at points *l* and *m*, the threshold value $th^{\left(lm\right)}$ is defined as:

(25) $$th^{\left(lm\right)}=\sum_{j=1}^{l}∥S_{wl}^{\left(j\right)}{∥}_{\infty }w_{M}^{\left(j\right)}+\sum_{j=l+1}^{m}{∥S_{wm}^{\left(j\right)}∥}_{\infty }w_{M}^{\left(j\right)}$$

Then, by considering the measurement noise, Algorithm 1 is updated into Algorithm 2 as follows:
**Algorithm 2** Fault isolation algorithm in the presence of measurement noise.Measure $y_{fnss}^{\left(l\right)}$ and $y_{fnss}^{\left(m\right)}$ in steady state.Compute $y_{ss}^{\left(l\right)}$, $y_{ss}^{\left(m\right)}$, and $th^{\left(lm\right)}$.Isolate the fault:-If $∥y_{fnss}^{\left(l\right)}-y_{ss}^{\left(l\right)}∥-∥y_{fnss}^{\left(m\right)}-y_{ss}^{\left(m\right)}∥\in \left(-th^{\left(lm\right)},th^{\left(lm\right)}\right)$, then no fault event occurred or the fault is negligible in comparison to the threshold.-If $∥y_{fnss}^{\left(l\right)}-y_{ss}^{\left(l\right)}∥-∥y_{fnss}^{\left(m\right)}-y_{ss}^{\left(m\right)}∥>th^{\left(lm\right)}$, then the fault occurred before *l* and after *m*.-If $∥y_{fnss}^{\left(l\right)}-y_{ss}^{\left(l\right)}∥-∥y_{fnss}^{\left(m\right)}-y_{ss}^{\left(m\right)}∥<-th^{\left(lm\right)}$, then the fault occurred before *m* and after *l*.

**Example 3.**  *This example is an extension of Example 2.*

*Consider that in the loop shown in Figure 4, the subsystem’s sensors are affected by measurement noises* $w^{\left(P\right)}$ *and* $w^{\left(Q\right)}$*, where* ${∥w^{\left(P\right)}\left(t\right)∥}_{\infty }<w_{M}^{\left(P\right)}$ *and* ${∥w^{\left(Q\right)}\left(t\right)∥}_{\infty }<w_{M}^{\left(Q\right)}$*.*

*Then, using Equations *(24)* and *(25)*, the threshold value can be computed as follows:* 

(26) $$th^{\left(PQ\right)}={∥\frac{1-{∥S_{1}^{\left(Q\right)}∥}_{0}}{1-∥S_{1}^{\left(P\right)}{∥}_{0}{∥S_{1}^{\left(Q\right)}∥}_{0}}∥}_{\infty }w_{M}^{\left(P\right)}+{∥\frac{{∥S_{1}^{\left(P\right)}∥}_{0}-1}{1-∥S_{1}^{\left(P\right)}{∥}_{0}{∥S_{1}^{\left(Q\right)}∥}_{0}}∥}_{\infty }w_{M}^{\left(Q\right)}$$

### 4.4. Fault Estimation

After the fault has been isolated, under certain sensor placement assumptions, the fault estimation can be performed. A linear disturbance–observer approach is proposed to determine the magnitude of the fault given by Equation (7).

During the estimation process, consider k=l=1 and the fault has been isolated in subsystem k=1.

The state space model given by Equation (1) with fault (7) can be rewritten as follows:

(27) $$\begin{array}{c} \begin{array}{cc} {\dot{x}}^{\left(k\right)} & =A^{\left(k\right)}x^{\left(k\right)}+B^{\left(k\right)}u_{X}^{\left(k\right)}+E^{\left(k\right)}f^{\left(k\right)}\\ y^{\left(k\right)} & =Cx^{\left(k\right)}\\ E^{\left(k\right)} & ={\left[\begin{array}{ccccccc} k_{E}^{\left(k\right)} & k_{E}^{\left(k\right)} & \ldots & k_{E}^{\left(k\right)} & \ldots & k_{E}^{\left(k\right)} & k_{E}^{\left(k\right)} \end{array}\right]}^{T} \end{array} \end{array}$$

where $E^{\left(k\right)}$ is a fault distribution column matrix and $f^{\left(k\right)}$ is assumed to be constant.

This new model in Equation (27) can be further transformed into an extended state space model by defining an extended state vector containing the fault as $z^{\left(k\right)}={\left[x^{\left(k\right)}\;f^{\left(k\right)}\right]}^{T}$:

(28) $$\begin{array}{c} \begin{array}{cc} {\dot{z}}^{\left(k\right)} & =\left[\begin{array}{cc} A^{\left(k\right)} & E^{\left(k\right)}\\ 0 & 0 \end{array}\right]\left[\begin{array}{c} x^{\left(k\right)}\\ f^{\left(k\right)} \end{array}\right]+\left[\begin{array}{c} B^{\left(k\right)}\\ 0 \end{array}\right]u_{X}^{\left(k\right)}=A_{z}^{\left(k\right)}z^{\left(k\right)}+B_{z}^{\left(k\right)}u_{X}^{\left(k\right)}\\ y^{\left(k\right)} & =\left[\begin{array}{cc} C & 0 \end{array}\right]\left[\begin{array}{c} x^{\left(k\right)}\\ f^{\left(k\right)} \end{array}\right]=C_{z}z^{\left(k\right)} \end{array} \end{array}$$

As stated by [34], if the steady state value of the fault is not zero, a proportional observer cannot correctly estimate the states of the plant because there will always be a steady state error between the actual and estimated states. However, using a proportional-integral (PI) observer, the steady state error can be reduced.

The state space model of a PI observer is:

(29) $$\begin{array}{c} \begin{array}{cc} {\dot{\hat{x}}}^{\left(k\right)} & =A^{\left(k\right)}{\hat{x}}^{\left(k\right)}+B^{\left(k\right)}u_{X}^{\left(k\right)}+L_{P}^{\left(k\right)}\left(y^{\left(k\right)}-{\hat{y}}^{\left(k\right)}\right)+E^{\left(k\right)}{\hat{f}}^{\left(k\right)}\\ {\dot{\hat{f}}}^{\left(k\right)} & =L_{I}^{\left(k\right)}\left(y^{\left(k\right)}-{\hat{y}}^{\left(k\right)}\right)\\ {\hat{y}}^{\left(k\right)} & =C{\hat{x}}^{\left(k\right)} \end{array} \end{array}$$

where ${\hat{x}}^{\left(k\right)}$ is the estimated state vector, ${\hat{f}}^{\left(k\right)}$ is the estimated fault magnitude, $L_{P}^{\left(k\right)}$ is the observer’s proportional gain, and $L_{I}^{\left(k\right)}$ is the observer’s integral gain.

Hence, a PI observer can be designed for the extended state space model in Equation (28) to not only estimate the states but also the fault. By applying Equation (29) into Equation (28) with the assumption that $\left(A_{z}^{\left(k\right)},C_{z}\right)$ is an observable pair, we obtain:

(30) $$\begin{array}{c} \begin{array}{cc} {\dot{\hat{z}}}^{\left(k\right)} & =\left(\left[\begin{array}{cc} A^{\left(k\right)} & E^{\left(k\right)}\\ 0 & 0 \end{array}\right]-\left[\begin{array}{c} L_{P}^{\left(k\right)}\\ L_{I}^{\left(k\right)} \end{array}\right]\left[\begin{array}{cc} C & 0 \end{array}\right]\right){\hat{z}}^{\left(k\right)}+\left[\begin{array}{c} L_{P}^{\left(k\right)}\\ L_{I}^{\left(k\right)} \end{array}\right]y^{\left(k\right)}+\left[\begin{array}{c} B^{\left(k\right)}\\ 0 \end{array}\right]u_{X}^{\left(k\right)}\\ & =\left(A_{z}^{\left(k\right)}-L_{z}^{\left(k\right)}C_{z}\right){\hat{z}}^{\left(k\right)}+L_{z}^{\left(k\right)}y^{\left(k\right)}+B_{z}^{\left(k\right)}u_{X}^{\left(k\right)}\\ {\hat{y}}^{\left(k\right)} & =C_{z}{\hat{z}}^{\left(k\right)} \end{array} \end{array}$$

Thus, if $L_{P}^{\left(k\right)}$ and $L_{I}^{\left(k\right)}$ are chosen such that $\left(A_{z}^{\left(k\right)}-L_{z}^{\left(k\right)}C_{z}\right)$ is Hurwitz, then $\lim_{t\to \infty }\left(z^{\left(k\right)}-{\hat{z}}^{\left(k\right)}\right)=0$.

Meanwhile, in the presence of noise, the widely known linear quadratic estimator (LQE) procedure can be used to compute the observer’s gain $L_{z}^{\left(k\right)}$. For details, see Appendix A.

## 5. Case Studies

To verify and validate the proposed fault isolation and fault estimation methods, two simulation case studies were examined in the MATLAB/Simulink environment. The first case study is based on Example 2. Meanwhile, for the second case study, the investigated network comprises six subsystems as presented in Example 1.

### 5.1. Case Study 1

In this case study, two identical subsystems $S^{\left(P\right)}$ and $S^{\left(Q\right)}$ are connected as shown in Figure 4. The model of the subsystems is given by Equation (20) where each subsystem is considered to have five state variables (n=5). The applied parameters and external inputs are shown in Table 1.

It is considered that the measurements $y^{\left(P\right)}$ and $y^{\left(Q\right)}$ are influenced by noise as presented in (20). To compensate for the noise that influences the system, a suitable threshold $th^{\left(PQ\right)}$ is computed by applying Equation (25). From the proposed fault isolation logic (see Algorithm 2), as the loop is composed of two subsystems $S^{\left(P\right)}$ and $S^{\left(P\right)}$, the simulation results should show that $∥y_{fnss}^{\left(P\right)}-y_{ss}^{\left(P\right)}∥-∥y_{fnss}^{\left(Q\right)}-y_{ss}^{\left(Q\right)}∥$ is above $th^{\left(PQ\right)}$ when there is a fault in subsystem $S^{\left(P\right)}$, or $∥y_{fnss}^{\left(P\right)}-y_{ss}^{\left(P\right)}∥-∥y_{fnss}^{\left(Q\right)}-y_{ss}^{\left(Q\right)}∥$ is below $-th^{\left(PQ\right)}$ when there is a fault in subsystem $S^{\left(Q\right)}$.

**Figure 5:**
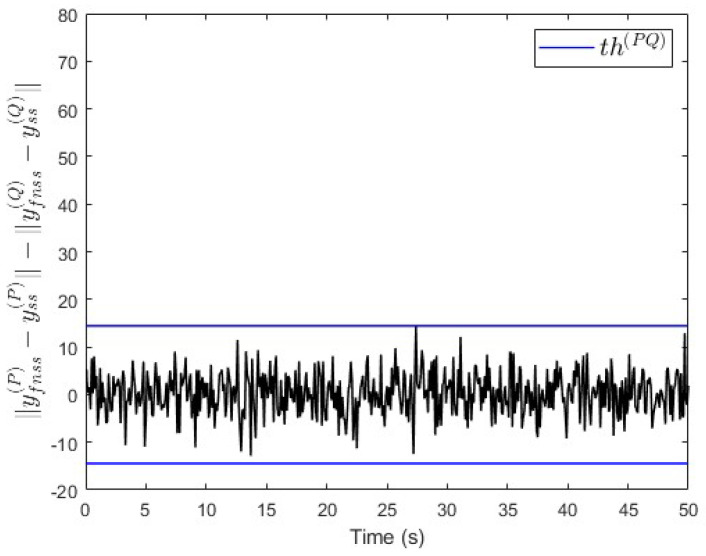
Case study 1—fault free case.

In the first scenario, no fault is injected into the subsystems. The simulation results in Figure 5 show $∥y_{fnss}^{\left(P\right)}-y_{ss}^{\left(P\right)}∥-∥y_{fnss}^{\left(Q\right)}-y_{ss}^{\left(Q\right)}∥\in \left(-th^{\left(PQ\right)},th^{\left(PQ\right)}\right)$, i.e., no fault is injected.

**Figure 6:**
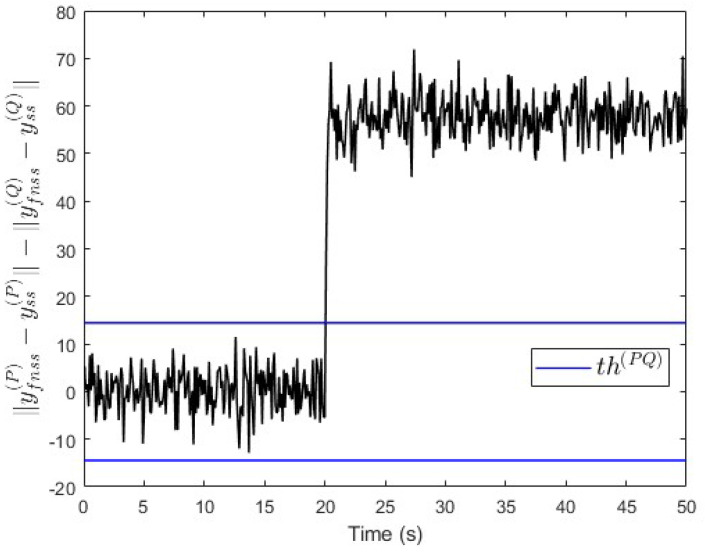
Case study 1—fault isolation in subsystem $S^{\left(P\right)}$.

In the second scenario, a constant fault signal $f^{\left(P\right)}=100$ is injected into subsystem $S^{\left(P\right)}$ at t=20 s. Figure 6 shows that the value of $∥y_{fnss}^{\left(P\right)}-y_{ss}^{\left(P\right)}∥-∥y_{fnss}^{\left(Q\right)}-y_{ss}^{\left(Q\right)}∥$ is above the threshold after the fault event occurred. This indicates that a fault is occurring in subsystem $S^{\left(P\right)}$.

**Figure 7:**
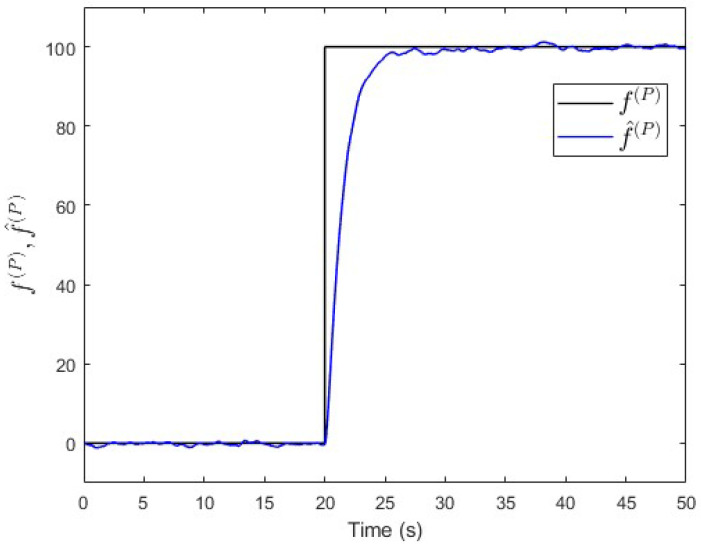
Case study 1—fault estimation in subsystem $S^{\left(P\right)}$.

**Figure 8:**
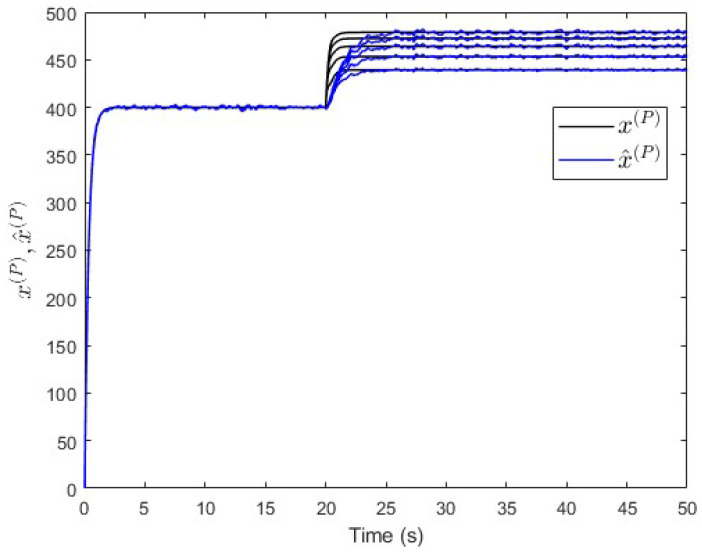
Case study 1—states estimation in subsystem $S^{\left(P\right)}$.

To verify the proposed fault estimation approach, an observer is designed specifically for subsystem $S^{\left(P\right)}$ based on Equation (30). By using the lqe MATLAB function (see Appendix A), the observer’s gain is computed such that the measurement noise variances are taken into consideration. Figure 7 shows the fault and its estimated value. It is seen that the fault’s magnitude is correctly estimated. Meanwhile, Figure 8 shows that the observer successfully estimates the states of subsystem $S^{\left(P\right)}$.

**Figure 9:**
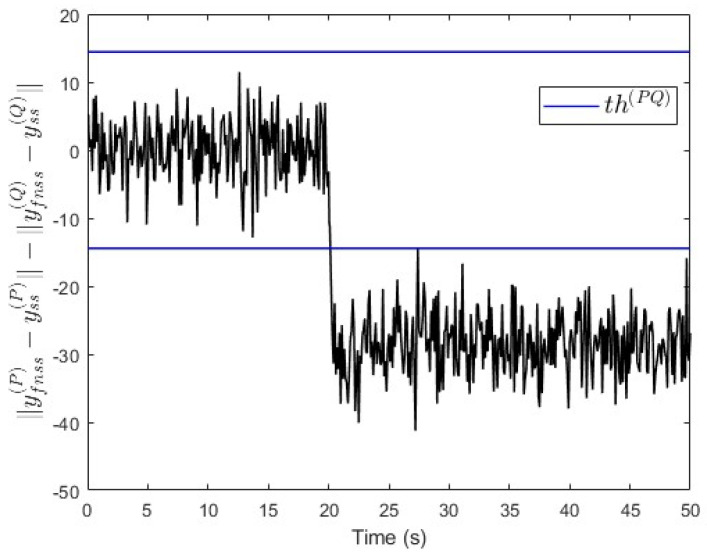
Case study 1—fault isolation in subsystem $S^{\left(Q\right)}$.

In the third scenario, a constant fault signal $f^{\left(Q\right)}=-50$ is injected into subsystem $S^{\left(Q\right)}$ at t=20 s. Figure 9 shows that the value of $∥y_{fnss}^{\left(P\right)}-y_{ss}^{\left(P\right)}∥-∥y_{fnss}^{\left(Q\right)}-y_{ss}^{\left(Q\right)}∥$ is below $-th^{\left(PQ\right)}$ after the fault event occurred. This indicates that a fault is occurring in subsystem $S^{\left(Q\right)}$, which is correct.

**Figure 10:**
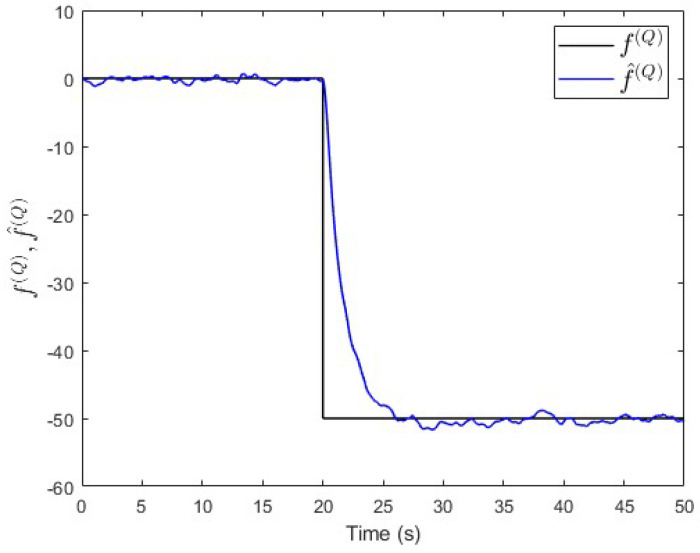
Case study 1—fault estimation in subsystem $S^{\left(Q\right)}$.

**Figure 11:**
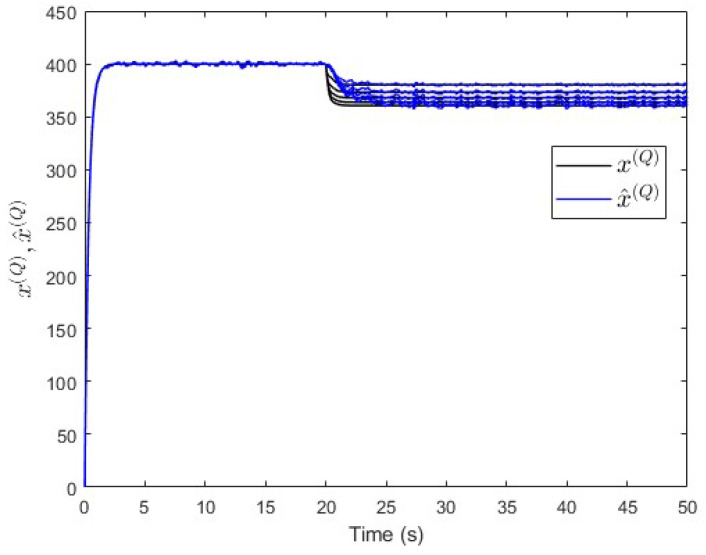
Case study 1—states estimation in subsystem $S^{\left(Q\right)}$.

Moreover, an observer is designed specifically for subsystem $S^{\left(Q\right)}$ based on Equation (30). The observer’s gain is also computed using the same lqe MATLAB function such that the noise variances are taken into consideration. Figure 10 shows the fault and its estimated value. Here, the fault’s magnitude is also correctly estimated. Meanwhile, Figure 11 shows that the observer successfully estimates the states of subsystem $S^{\left(Q\right)}$.

### 5.2. Robustness Analysis (for Case Study 1)

To analyse the robustness of our proposed approach, modified parameter values are considered in the subsystem model (1) only during the simulations. Here, we have two parameters to modify: the mass flow rate and the transfer coefficient. The mass flow rate $v^{\left(j\right)}$ can be easily measured. However, the transfer coefficient $k_{E}^{\left(j\right)}$ can only be estimated as its value changes depending on the physical conditions and circumstances. Thus, the robustness analysis is performed by checking the performance of fault isolation and estimation in the presence of transfer coefficient parameter uncertainty.

**Figure 12:**
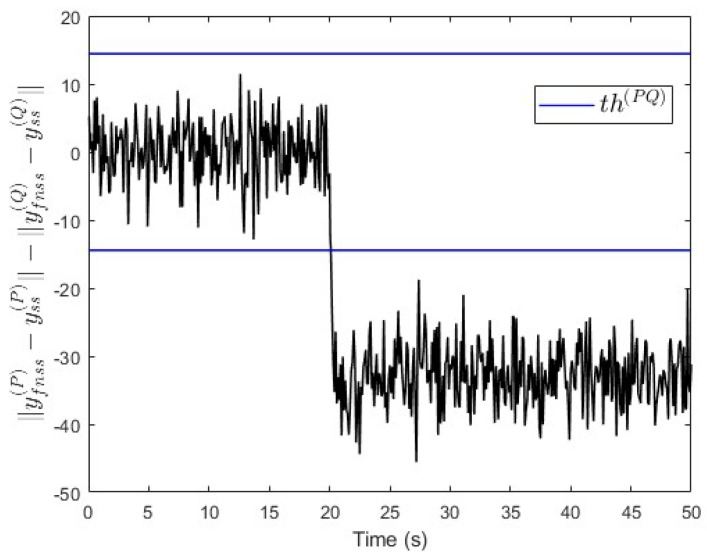
Case study 1—fault isolation in subsystem $S^{\left(Q\right)}$ with a parameter change.

**Figure 13:**
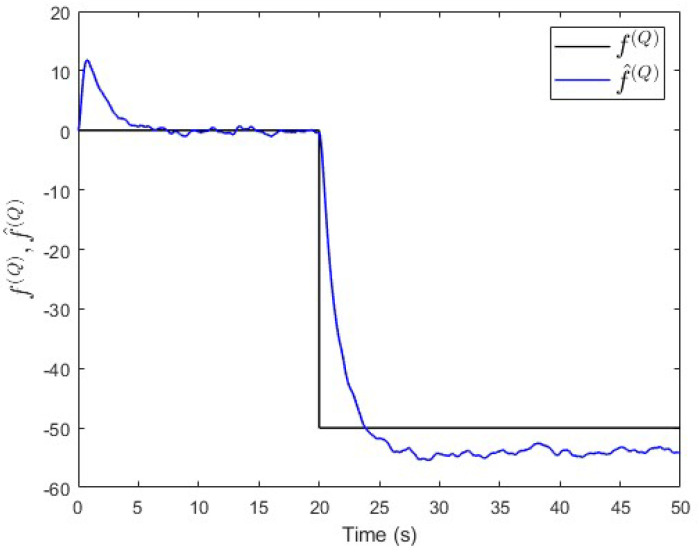
Case study 1—fault estimation in subsystem $S^{\left(Q\right)}$ with a parameter change.

By using case study 1 for the sake of convenience but without loss of generality, the simulation is carried out by increasing the value of $k_{E}^{\left(j\right)}$ in Table 1 by 25% ($k_{E}^{\left(j\right)}=3.75$ where j=P,Q). After this, a constant fault signal $f^{\left(Q\right)}=-50$ is injected into subsystem $S^{\left(Q\right)}$ at t=20 s. Figure 12 shows that the value of $∥y_{fnss}^{\left(P\right)}-y_{ss}^{\left(P\right)}∥-∥y_{fnss}^{\left(Q\right)}-y_{ss}^{\left(Q\right)}∥$ is below $-th^{\left(PQ\right)}$ after the fault event occurred. This indicates that a fault is occurring in subsystem $S^{\left(Q\right)}$, which is correct. The real and estimated fault values are shown in Figure 13. There, the fault’s magnitude is estimated with an error of less than 10%. This error is acceptable provided that the maximum tolerable value of the uncertainty in $k_{E}^{\left(j\right)}$ is 25%.

### 5.3. Incipient Fault Analysis (for Case Study 1)

**Figure 14:**
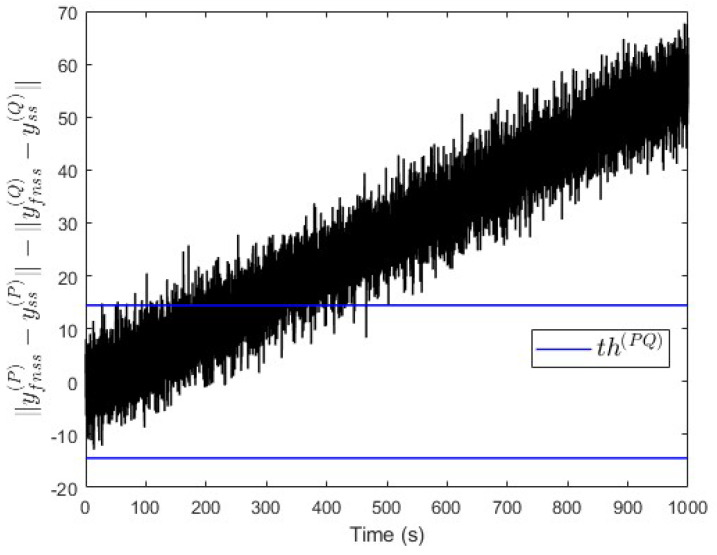
Case study 1—fault isolation in subsystem $S^{\left(P\right)}$ with an incipient fault.

**Figure 15:**
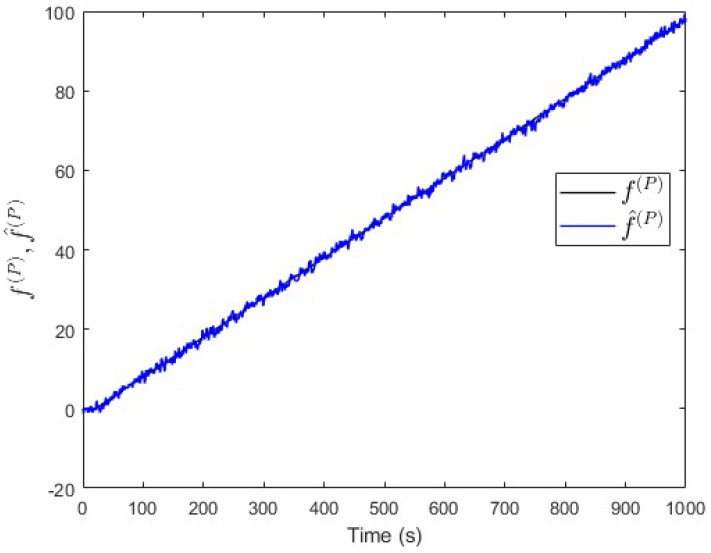
Case study 1—fault estimation in subsystem $S^{\left(P\right)}$ with an incipient fault.

We also analysed the performance of our proposed approach in the case of an incipient fault (slowly developing fault). To do this, by using case study 1 again, a linearly increasing fault signal is assumed with an initial value of 0 and a slope value of 0.1 per unit of time. This fault is injected into subsystem $S^{\left(P\right)}$ at t=20 s. Figure 14 shows that the value of $∥y_{fnss}^{\left(P\right)}-y_{ss}^{\left(P\right)}∥-∥y_{fnss}^{\left(Q\right)}-y_{ss}^{\left(Q\right)}∥$ is above the threshold $th^{\left(PQ\right)}$ some time after the fault event occurred. This indicates that a fault is occurring in subsystem $S^{\left(P\right)}$, which is correct. Meanwhile, Figure 15 shows that the observer successfully estimates the magnitude of the incipient fault.

### 5.4. Case Study 2

In this case study, six subsystems $S^{\left(A\right)}$, $S^{\left(B\right)}$, $S^{\left(C\right)}$, $S^{\left(D\right)}$, $S^{\left(E\right)}$, and $S^{\left(F\right)}$ are connected as shown in Figure 1. The model of the subsystems is given by Equation (20) where each subsystem is considered to have five state variables (n=5). The applied parameters and external inputs are shown in Table 2.

In this network, two sensors are placed to measure both $y^{\left(A\right)}$ and $y^{\left(F\right)}$ that are affected by noise as presented in (20). To compensate for the noise that influences the system, a suitable threshold $th^{\left(AF\right)}$ is computed as presented in Equation (25). As both subsystem $S^{\left(A\right)}$ and $S^{\left(F\right)}$ are inside the loops of either $\left\{S^{\left(A\right)},S^{\left(B\right)},S^{\left(D\right)},S^{\left(F\right)}\right\}$ or $\left\{S^{\left(A\right)},S^{\left(B\right)},S^{\left(E\right)},S^{\left(F\right)}\right\}$, the simulation’s results should show that: $∥y_{fnss}^{\left(A\right)}-y_{ss}^{\left(A\right)}∥-∥y_{fnss}^{\left(F\right)}-y_{ss}^{\left(F\right)}∥$ is above the threshold $th^{\left(AF\right)}$ when there is a fault in subsystem $S^{\left(A\right)}$, or $∥y_{fnss}^{\left(A\right)}-y_{ss}^{\left(A\right)}∥-∥y_{fnss}^{\left(F\right)}-y_{ss}^{\left(F\right)}∥$ is below $-th^{\left(AF\right)}$ when there is a fault in either subsystem $S^{\left(B\right)}$, $S^{\left(C\right)}$, $S^{\left(D\right)}$, $S^{\left(E\right)}$, or $S^{\left(F\right)}$ (see Algorithm 2).

**Figure 16:**
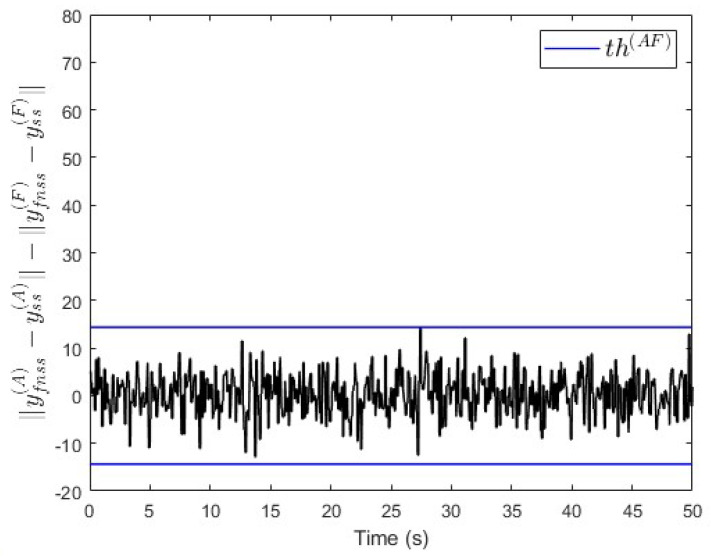
Case study 2—fault free case.

In the first scenario, no fault is injected into the subsystems. The simulation results in Figure 16 show that $∥y_{fnss}^{\left(A\right)}-y_{ss}^{\left(A\right)}∥-∥y_{fnss}^{\left(F\right)}-y_{ss}^{\left(F\right)}∥\in \left(-th^{\left(AF\right)},th^{\left(AF\right)}\right)$, i.e., no fault is injected.

**Figure 17:**
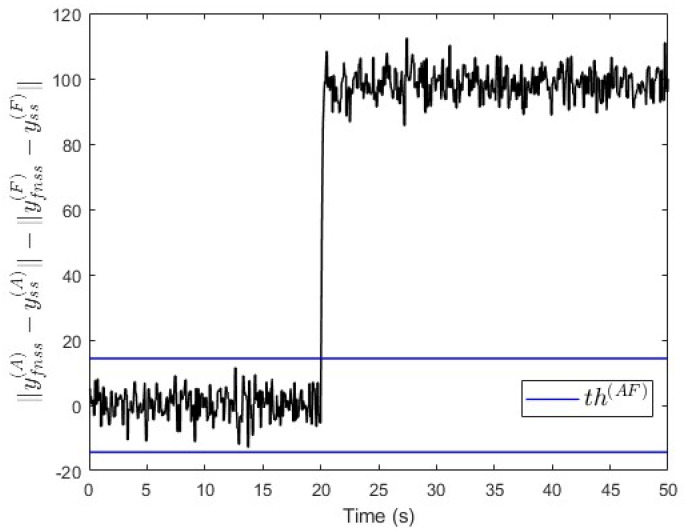
Case study 2—fault isolation in subsystem $S^{\left(A\right)}$.

In the second scenario, a constant fault signal $f^{\left(A\right)}=200$ is injected into subsystem $S^{\left(A\right)}$ at t=20 s. Figure 17 shows that the value of $∥y_{fnss}^{\left(A\right)}-y_{ss}^{\left(A\right)}∥-∥y_{fnss}^{\left(F\right)}-y_{ss}^{\left(F\right)}∥$ is above the threshold after the fault event occurred. This indicates that a fault is occurring in subsystem $S^{\left(A\right)}$, which is correct.

**Figure 18:**
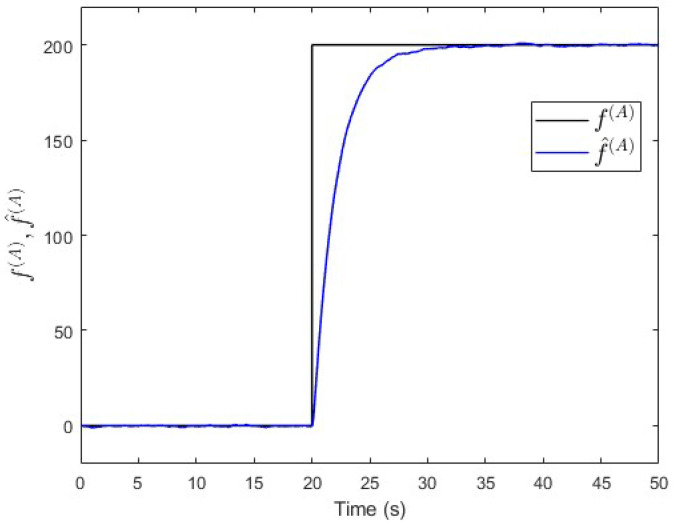
Case study 2—fault estimation in subsystem $S^{\left(A\right)}$.

**Figure 19:**
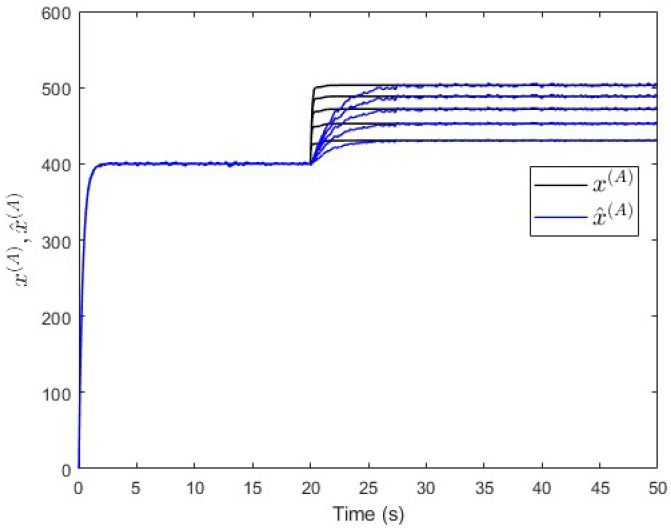
Case study 2—states estimation in subsystem $S^{\left(A\right)}$.

To verify the proposed fault estimation approach, an observer is designed for subsystem $S^{\left(A\right)}$ based on Equation (30). By using the lqe MATLAB function, the observer’s gain is computed such that the measurement noise variances are taken into consideration. Figure 18 shows the fault and its estimated value. It is seen that the fault’s magnitude is correctly estimated. Meanwhile, Figure 19 shows that the observer successfully estimates the states of subsystem $S^{\left(A\right)}$.

**Figure 20:**
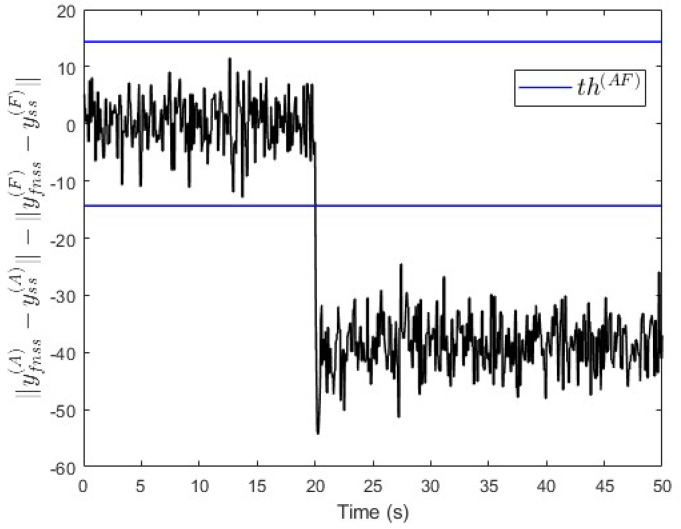
Case study 2—fault isolation in subsystem $S^{\left(F\right)}$.

In the third scenario, a constant fault signal $f^{\left(F\right)}=-150$ is injected into subsystem $S^{\left(F\right)}$ at t=20 s. Figure 20 shows that the value of $∥y_{fnss}^{\left(A\right)}-y_{ss}^{\left(A\right)}∥-∥y_{fnss}^{\left(F\right)}-y_{ss}^{\left(F\right)}∥$ is below $-th^{\left(AF\right)}$ after the fault event occurred. This indicates that a fault is occurring either in subsystem $S^{\left(B\right)}$, $S^{\left(C\right)}$, $S^{\left(D\right)}$, $S^{\left(E\right)}$, or $S^{\left(F\right)}$, which is correct.

**Figure 21:**
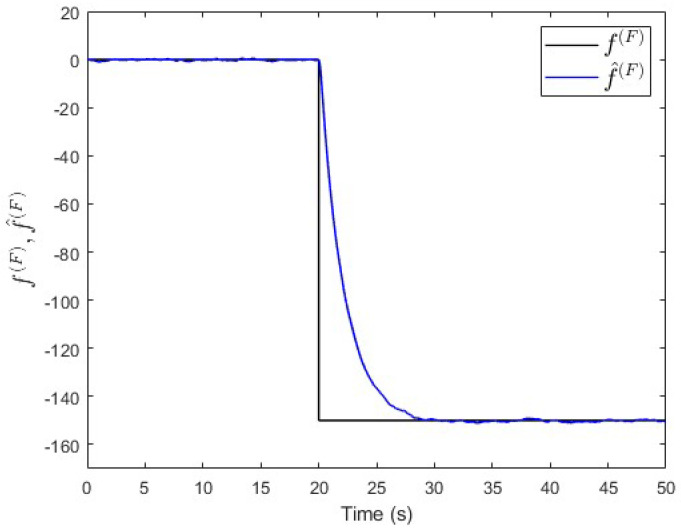
Case study 2—fault estimation in subsystem $S^{\left(F\right)}$.

**Figure 22:**
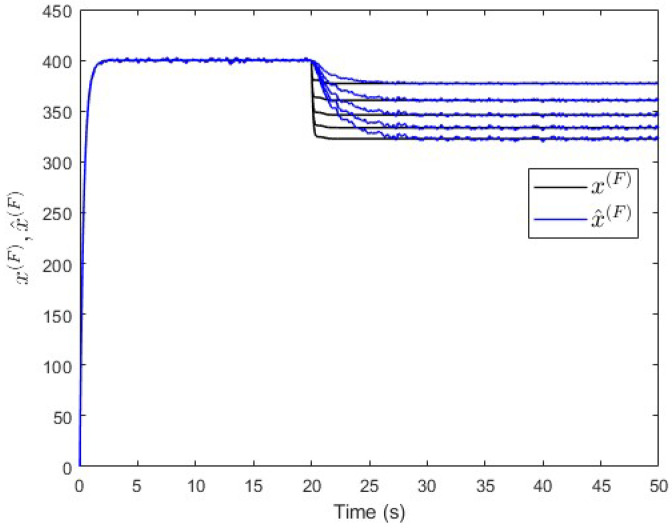
Case study 2—states estimation in subsystem $S^{\left(F\right)}$.

Moreover, an observer is designed specifically for subsystem $S^{\left(F\right)}$ based on the same Equation (30). The observer’s gain is computed using the same lqe MATLAB function such that the noise variances are taken into consideration. Figure 21 shows the fault and its estimated value. Here, the fault’s magnitude is also correctly estimated. Meanwhile, Figure 22 shows that the observer successfully estimates the states of subsystem $S^{\left(F\right)}$.

## 6. Conclusions

The case of networked linear process systems is considered in this paper with only a single conserved extensive quantity but with a network structure containing loops. It is assumed that the probability of multiple faults happening at the same time in the network is negligible.

For fault detection and isolation purposes, the network elements are described by a simple 2ISO LTI state space model where the fault enters as an additive linear term into the second input of the equations. Using the models of the network elements, a general model of the network is constructed which includes static splitting and joining connections. This results in an LTI state space model for the overall system that is suitable for fault detection and isolation.

By analysing the effect of the fault in a subsystem that propagates to the sensors’ measurements at different positions, a fault isolation algorithm (Algorithm 1) was proposed. It uses two sensors, installed at the output of the two subsystems placed along a path, to locate the fault (i.e., decide if the fault has occurred in a subsystem on the connecting path or outside it). A steady state analysis and superposition principle were used to build the algorithm. An improved version of the algorithm (Algorithm 2) was also proposed to perform the localization in the presence of measurement noise.

Having completed the fault isolation process, a PI-based disturbance observer was then proposed to estimate the magnitude of the fault.

Two simulation case studies were used in the MATLAB/Simulink environment to verify and validate the proposed fault isolation and fault estimation methods. The first case study comprised a loop with two subsystems only. Meanwhile, the investigated network of the second case study comprised six subsystems. Separate subsections on analysing the parametric robustness and effect of an incipient fault were provided using the first case study for the sake of convenience but without generality loss.

In both case studies, the simulation results show good performance both in fault detection and isolation, and in fault magnitude estimation. In addition, our proposed method also shows good robustness against the transfer coefficient $k_{E}$ which is difficult to estimate in practice. By using a ramp-like fault signal as an incipient fault, the simulation results show that our proposed fault estimation method can correctly estimate its magnitude.

Further work includes the extension of our method to cases of multiple faults, and verifying and validating the method on real data of, e.g., household heating/cooling systems.

## Figures and Tables

**Table 1:** Case study 1—parameters and external inputs.

*j*	*P*	*Q*
$v^{\left(j\right)}$	10	10
$k_{E}^{\left(j\right)}$	3	3
$u_{E}^{\left(j\right)}$	400	400
$R_{u}^{\left(j\right)}$	20	20
$Q_{y}^{\left(j\right)}$	20	20

**Table 2:** Case study 2—parameters and external inputs.

*j*	*A*	*B*	*C*	*D*	*E*	*F*
$v^{\left(j\right)}$	20	4	16	8	12	20
$k_{E}^{\left(j\right)}$	3	3	3	3	3	3
$R_{u}^{\left(j\right)}$	20	20	20	20	20	20
$Q_{y}^{\left(j\right)}$	20	20	20	20	20	20

## Data Availability

Data will be made available on request.

## Appendix A. Linear Quadratic Estimator (LQE)

Consider an extended state space model, as in Equation (28), is influenced by noise inputs as follows:

(A1) $$\begin{array}{c} \begin{array}{cc} {\dot{z}}^{\left(k\right)} & =A_{z}^{\left(k\right)}z^{\left(k\right)}+B_{z}^{\left(k\right)}u_{X}^{\left(k\right)}+G_{z}^{\left(k\right)}w_{uX}^{\left(k\right)}\\ y^{\left(k\right)} & =C_{z}z^{\left(k\right)}+w_{y}^{\left(k\right)} \end{array} \end{array}$$

where $G_{z}^{\left(k\right)}$ is the noise distribution matrix. In this system class, $G_{z}^{\left(k\right)}=B_{z}^{\left(k\right)}$.

Furthermore, assume that $w_{uX}^{\left(k\right)}$ and $w_{y}^{\left(k\right)}$ are uncorrelated white noise with variance: $w_{uX}^{\left(k\right)}\left(t\right)∼\left(0,R_{u}^{\left(k\right)}\right),\;w_{y}^{\left(k\right)}\left(t\right)∼\left(0,Q_{y}^{\left(k\right)}\right)$.

Now, a state estimation error covariance matrix $P^{\left(k\right)}$ is defined that satisfies:

(A2) $$\begin{array}{c} \begin{array}{cc} P^{\left(k\right)} & ≻0\\ {\dot{P}}^{\left(k\right)} & =A_{z}^{\left(k\right)}P^{\left(k\right)}+P^{\left(k\right)}{\left(A_{z}^{\left(k\right)}\right)}^{T}+G_{z}^{\left(k\right)}R_{u}^{\left(k\right)}{\left(G_{z}^{\left(k\right)}\right)}^{T}-P^{\left(k\right)}C_{z}^{T}{\left(Q_{y}^{\left(k\right)}\right)}^{-1}C_{z}P^{\left(k\right)} \end{array} \end{array}$$

As expected, the error covariance $P^{\left(k\right)}$ diminishes quickly in a steady state value (${\dot{P}}^{\left(k\right)}=0$); therefore, the following Riccati equation can be solved:

(A3) $$0=A_{z}^{\left(k\right)}P^{\left(k\right)}+P^{\left(k\right)}{\left(A_{z}^{\left(k\right)}\right)}^{T}+G_{z}^{\left(k\right)}R_{u}^{\left(k\right)}{\left(G_{z}^{\left(k\right)}\right)}^{T}-P^{\left(k\right)}C_{z}^{T}{\left(Q_{y}^{\left(k\right)}\right)}^{-1}C_{z}P^{\left(k\right)}$$

Then, the observer gain $L_{z}^{\left(k\right)}$ can be computed as [35,36]:

(A4) $$L_{z}^{\left(k\right)}=P^{\left(k\right)}C_{z}^{T}{\left(Q_{y}^{\left(k\right)}\right)}^{-1}$$

The gain $L_{z}^{\left(k\right)}$ in (A4) can be directly obtained by using the MATLAB function ‘lqe’.
